# Malformations of Cortical Development, Cognitive Involvementand Epilepsy: A Single Institution Experience in 19 Young Patients

**DOI:** 10.3390/children8080637

**Published:** 2021-07-26

**Authors:** Valeria Venti, Maria Chiara Consentino, Pierluigi Smilari, Filippo Greco, Claudia Francesca Oliva, Agata Fiumara, Raffaele Falsaperla, Martino Ruggieri, Piero Pavone

**Affiliations:** 1 Section of Pediatrics and Child Neuropsychiatry, Department of Clinical and Experimental Medicine, University of Catania, Via S. Sofia 78, 95123 Catania, Italy; ventivaleria1@gmail.com (V.V.); smilari@policlinico.it (P.S.); coicoico@hotmail.com (F.G.); agatafiumara@yahoo.it (A.F.); m.ruggieri@unict.it (M.R.); 2 Postgraduate Training Program in Pediatrics, Department of Clinical and Experimental Medicine, University of Catania, 95123 Catania, Italy; mchiara.consentino@gmail.com (M.C.C.); claudiaf.oliva@gmail.com (C.F.O.); 3 Unit of Pediatrics and Neonatology, Neonatal Intensive Care, and Pediatric Emergency, AUO San Marco-Policlinico, University of Catania, 95123 Catania, Italy; raffaelefalsaperla@hotmail.com

**Keywords:** epilepsy, cortical development, developmental delay

## Abstract

Background. Malformations of cortical development (MCD) include a wide range of congenital disorders mostly causing severe cognitive dysfunction and epilepsy. Objective: to report on clinical features including cognitive involvement, epileptic seizures with response to antiseizure medications, comorbidities in young patients affected by MCD and followed in a single tertiary hospital. Patients and methods: A retrospective review of the medical records and magnetic resonance images (MRI) of 19 young patients with an age ranging between eight days and fifteen years affected by MCD and admitted to Pediatrics Department University of Catania, Italy from October 2009 and October 2020 were selected. Patients were distinguished in three groups following the Barcovich et al. 2012 classification for MCD: 4 (21%) in Group I; 8 (42%) in Group II; and, and 7 (37%) in Group III. Clinical features and MRI of the patients including cognitive involvement, epilepsy type and response to drugs treatment were analyzed. Results: In Group I, two patients showed cortical dysplasia and two dysembryoplastic neuroepithelial tumors plus focal cortical dysplasia; developmental delay/intellectual disability (DD/ID) was severe in one, moderate in one and absent in two; the type of seizures was in all the cases focal to bilateral tonic-clonic (FBTCs), and drug resistant was found in one case. In Group II, three patients showed neuronal hetero-topias and five had pachygyria-lissencephaly: DD/ID was severe in four, moderate in two, and absent in two; the type of seizure was focal (FS) in five, focal to bilateral tonic-clonic (FBTCs) in two, infantile spasms (IS) in one, and drug resistant was found in three. In Group III, six showed polymicrogyria and one schizencephaly: DD/ID was found severe in five, moderate in two, and the type of seizure was focal (FS) in five, FBTCS in two, and drug resistance was found in three.

## 1. Introduction

Malformations of cortical development (MCD) represent a wide and heterogeneous group of focal and diffuse lesions which occur during the cortical ontogenesis because of malfunctioning in its constitutive mechanisms. In 1996, Barkovich et al. [1] introduced a classification and definition of malformations of cortical development (MCD) in pediatric patients with developmental delay and epilepsy [1]. This classification has undergonesome changes in years, first in 2001, redefining categories basing on the stage of cortical development first affected [2]. In 2005, a further update has been proposed, more focused on genetic features as cause of MCD [3]. A specific clinical pathological focal cortical dysplasia (FCD) classification was proposed by the ILAE on 2010 on three tiered classification systems: FCD Type 1 to FCD Type III, on the basis of electroclinical presentation, imaging, neuropathological examination of surgical specimens, and postsurgical outcomes [4,5]. MCD has been distinguished in three groups: the first group, includes “malformations secondary to abnormal neuronal and glial proliferation or apoptosis”; the second group “malformations due to abnormal neuronal migration” (remained unmodified); the third group has changed his denomination from “malformations secondary to abnormal cortical organization” to “malformations secondary to abnormal post-migrational development”. The fourth group has been removed and its categories have been relocated [6]. MCD in its severe forms are characterized by notable developmental delay associated to early onset and intractable epileptic seizures. Although most of pediatric population with epilepsy seems to have a good response to antiseizure medications, around 15% of young patients develop a drug resistant epilepsy [7], and this is due in the 40% of cases [8,9,10] to cortical malformations. MCD with mild clinical manifestations are generally detected by instrumental exams performed after the first seizure episode. Magnetic resonance imaging (MRI) represents the essential diagnostic tool to identify and classify cortical malformations. In the last few years, molecular genetics and linkage analysis represent important instruments to identify disruptions in known involved genes which influence neuronal proliferation, migration, and subsequent cortical organization [11]. Recent reports have provided evidence that genetic mutations may influence not only MCD pattern but also cortical involvement localization [12,13,14]. The knowledge about the precise mechanism underlying epilepsy in MCD is still debated, some authors proposed a possible dysfunction in neurotransmitters as GABA or glutamate [15], or, for what concerns focal cortical dysplasia, an inadequate positioning of substratum and of radial glia-like cells which interfere on neuronal interconnection function, leading to electrical disturbances [16]. Epilepsy associated to MCD is frequent and, in a considerable number of patients, intractable, although the frequency and the type of seizure is different and very likely depending on the specific basal malformation [17,18,19,20]. ILAE 2010 guidelines defined epilepsy as drug resistant when is not possible to obtain a persistent and lasting seizure-free period, after treatment with two antiseizure medications well tolerated and appropriately chosen, individually or combined [18]. Drug-resistance in MCD may raise since epilepsy onset [21], or after years of good control, as it also might assume an intermittent trend [22,23]. Many studies have described how MCD are the cause of 25–40% of intractable epilepsies in children and young adults [6,7,8]. Some epileptic contexts previously classified as cryptogenic are currently associated to MCD and many studies highlighted its causal linkage [21]. We report, herewith, the results of a study carried out in 19 pediatric patients affected by MCD focusing on clinical features, including cognitive involvement, comorbidities, type of epilepsy and response to anti-epileptic drug treatment.

## 2. Materials and Methods

We performed a retrospective analysis, selecting patients with MCD observed at the Department of Pediatrics G.Rodolico University Hospital, Catania, Italy in a period between October 2009 and October 2020. The study was conducted ethically in accordance with the World Medical Association’s Declaration of Helsinki and was approved by the ethics committee of the University of Catania, Italy (Ethical Committee Catania Clinical Registration no. 97B/2020/PO). We included pediatric patients with MCD diagnosed by MRI and excluded those with DD/ID or epilepsy not associated to cortical malformations. ILAE 2017 classification has been used as clinical and nosographic reference to categorize seizure types and to define drug-resistance where occurred [17,18]. Pediatric patients with MCD have been subdivided into three group following the Barkovich 2012 classification [6]. We excluded from this study patients without clinical or EEG detailed study or with unknown pharmacological history. Nineteen young patients, with an age between 8 days to 15 years old (median age 2.9 years old) were enrolled. Nine of these young patients were males, ten were females. We excluded 6 patients that despite having MRI compatible with the diagnosis of MCD and neurologic features, had not developed seizures or EEG anomalies during their clinical history. All patients were evaluated during their hospitalization and during the follow up through accurate neurological examination. EEG recordings were obtained using a digital electroencephalograph) with brain explorer amplifier (EBNeuro-Galileo NT software (PMS database, Florence Italy), recorded at awake, hyperventilation, photic stimulation, during sleep and also through neuroimaging, and the Wechsler Intelligence Scale for children (WISC IV). Further assessments have been carried out on the basis of the underlying malformations and possible comorbidities, using laboratory tests, metabolic analysis and cerebrospinal fluid analysis. Genetic analysis was performed in 10 out of 19 patients: 5 of them have performed next generation sequencing (NGS) with evidence of VOUS (variant of uncertain significance) or likely pathogenetic variant that correlated with cases in literature with epilepsy and MCD (14); 4 patients performed array-CGH and one patient karyotype, telomer and MECP2 analysis with negative result for genomic rearrangements or mutations. Pharmacological response to antiseizure medications has been determined clinically and by EEG examination after three weeks from discharged and periodic follow-up established in relation to each patient, in order to detect the possible drug-resistance.

## 3. Results

Globally analyzing the clinical features in the three groups of patients (see Table 1), the cognitive impairment was the most common and reported feature, in 15 (78%) out of 19 patients, of moderate level in 5 and severe in 10. Moreover, one of these patients presented a severe behavioral disorder with aggressiveness which required treatment with neuroleptic. Only 4 patients showed normal cognitive level. Ten patients (52%) presented focal seizures, eight (42%) FBTCS and one (5%) IS. Two female patients with periventricular nodular heterotopia (PNH) and frontal polymicrogyria (PMG) presented congenital hypothyroidism with growth hormone (GH) deficiency under replacement therapy. Other comorbidities observed in this cohort were single case of ulcerative colitis, cortical blindness, kidney malformations, and hypospadias. MRI provided evidence of other cerebral malformations associated to MCD: 7 (36%) patients showed ventriculomegaly, 3 (15%) white matter periventricular calcifications, 2 (10%) had vermis hypoplasia and 1 (5%) had ponto-cerebellar hypoplasia. The therapeutic approach was different, depending on the heterogeneity of cortical malformations and their seizure types. In 11 (57%) patients, a single antiseizure medication was used, as levetiracetam (LEV), valproate (VPA), clonazepam (CLO), topiramate (TPA), lacosamide (LAC), phenobarbital (PHE). In 3 cases (15%), two drugs were used; in 4 (21%) patients, three drugs were used. In two children, treatment with ketogenic diet was started, but rapidly withdrawn due to poor compliance. None of the children were submitted to epilepsy surgery. In one patient, the antiseizure medication has been withdrawn one year after the child was seizure-free. Drug resistance was found in 7 (36%) patients. Epileptic children were considered pharmaco-resistant according to the definition of ILAE Commission [18] and the clinical conditions were established in the course of the disorder by PS, FG, AF, RF, PP, usually after a mutual opinion.

Results obtained in this cohort of patients were distinct in 3 groups following the Barkovich et al. classification [6]:

Group I includes 4 (21%) patients: 1 with bilateral temporo-parietal cortical dysplasia, 1 with parietal cortical dyplasia, and 2 with dysembrioplastic neuroepitelial tumors (DNET) plus focal cortical dysplasia; developmental delay/intellectual disability (DD/ID) was severe in 1, moderate in 1 and absent in 2, the type of seizure was in all the cases focal to bilateral tonic clonic (FBTCS), and drug resistance was found in 1 case.

Group II includes 8 (42%) patients; 2 with subcortical heterotopia, 1 with periventricular nodular heterotopia, 3 with lissencephaly, 1 with lissencephaly-pachygyrya, 1 with occipital pachygyrya; DD/ID was severe in 4, moderate in 2, and absent in 2, the type of seizure was focal (FS) in 5, FBTCS in 2, and infantile spams in 1. Drug resistance was found in 3 cases.

Group III includes 7 (37%) patients, 3 with bilateral frontal polymicrogyrya, 2 with bilateral fronto-parietal polymicrogyrya, 1 bilateral parieto-occipital microgyrya, 1 with unilateral schizencephaly; DD/ID was found severe in 5 patients, and moderate in 2, the type of seizure was FS in 5 and FBTCS in 2 cases, drug resistance was found in 3 cases. Results of genetic analysis were inconclusive.

## 4. Discussion

MCD are a heterogeneous group of complex disorders with the main clinical features being DD/ID and epilepsy with malformations often involving not only the brain but also other organs with related clinical manifestations. The heterogeneity of the clinical features presented by patients with MCD is confirmed by the results of this study. In this case—the series including a group of 19 young patients we found DD/ID in 15 cases (78%) and epileptic focal and FBTCS seizures in all the cases. A good response to anti-epileptic monotherapy was obtained in 12 (63%) of the cases (in Case 1, treatment was withdrawn after one year), while drug resistance was reported in 7 (36%). In addition to MCD other cerebral anomalies were found in 11 (54%). Aside the brain other body-organs were not affected apart from a case of renal ectopia and a case of hypospadias. To note, two patients presented with congenital hypothyroidism. Considering each of the three groups of MCD, in Group I of this study, two patients had cortical dysplasia (CD) and two patients showed DNET and focal cortical dysplasia (FCD). Both patients with FCD and DNET manifested the FBTCS seizures type. Leventer et al. [16] suggest that in cases with cortical dysplasia, epilepsy might present with different features based on the site of the cortical dysplasia and on the age of the patient. In this case-series the age of children was very young for both (three and two years, respectively), localization was temporo-parietal in one and parietal in the other, seizure types were similar but severity of cognitive impairment and resistance to treatment were different, more severe in one of the child and less in the other. In these disorders, different factors may concur to cause different clinical expression. There is evidence in patients with FCD of cases of milder course in front to cases of intractable and life-threatening epilepsy [16,22]. Dysembryoplastic neuroepithelial tumor (DNET) is a benign glioneuronal neoplasm that most commonly affects children manifesting with seizures often drug resistant [23]. In the present study both the children presently show a favorable response to drug treatment. Analyzing the clinical features of the patients belonging to Group II with diagnosis of periventricular-nodular heterotopia (PNH) and subcortical heterotopia, a clinical variability was noted: regarding the age at onset, six of the children presented the first seizure during his first year of life, while other children of the same group expressed symptoms during their second decade of age. Such data complies with evidence reported from Barkovich et al. [21], who report that epilepsy might occur at any age, and it is associated in most cases to ID, including speech delay, behavioral and movement disorders [24]. In the case-series FS type was the most common and drugs resistance was reported only in one case. In patients with lissencephaly, as described from Guerrini et al. [10], the age at onset of seizures in 90% of the cases is precocious and related to severe developmental delay with seizure onset within the first year of age; in the present cases the median age of seizure onset in cases of lissencephaly was seven months. In two children, at diagnosis, cerebral anomalies were detected as ponto-cerebellar hypoplasia and vermis hypoplasia: these features have been reported in patients with lissencephaly and mutations of RELN, coding for a protein that explicates its role on migrating cortical neurons by binding very low density lipoprotein (VLDRL) [25]. Among this case-series, only one patient with lissencephaly has performed next generation sequencing with evidence of a variant of uncertain significance identified in POMGNT2 and FAT4, respectively localized on chromosome 3p22.1 and 4q28.1, related to cobblestone lissencephaly, ventriculomegaly, cerebellar hypoplasia, ocular anomalies and Walker Warburg syndrome. The present patient had severe DD in association to ventriculomegaly and hypoplasic vermis. Group III involved 7 (56%) patients, 5 with polymicrogyria and 1 with schizencephaly. To note, two of them had congenital cytomegalovirus infection (CMV), and MRI showed also periventricular cerebral calcifications. Currently, hypoxia and cerebral hypoperfusion are also considered along with congenital CMV as non-genetic cause of polymicrogyria [24]. In this case series, an early onset age of seizures was noted with predominance of FS, with poor response to antiseizure medications, and severe developmental delay. Our findings emerge to agree with the results obtained by Rakade and Jensen (15), where the 83% of patients with schizencephaly presented moderate to severe developmental delay and onset of seizures around 13 months of age. In the case-series, 7 (36%) among the 19 MCD young patients complained by severe epilepsy and anti-epileptic drug resistance. Most of these MCD conditions are dominated by a significant number of seizure episodes before a proper start of treatment, and with an inadequate response to the first drug selected [20,26,27]. It is probable that these cases are determined by mechanism still not entirely understood, and possibly due to genetic discrepancies of carrier proteins structures and the consequent gap of transportation to the blood-brain barrier [28].

The limit of this study is mainly related to rather small sample of patients, lack of statistical analysis and genetic results. Only a small number of enrolled patients have undergone array-CGH and NGS with result of pathogenetic variants; many of the studies we reviewed reported a strong correlation among genetic mutations and disrupted neuronal migration associated to malformations of cortical development.

## Figures and Tables

**Table 1:** Clinical features of patients with MCD.

PT	Sex	MCD (MR)	Genetics	DD/ID	Seizure Onset Age	Type of Seizure	EEG	Comorbidities	Pregnancy	Past AEDs	Current AEDs	Drug-Resistance	Other Radiologic Features
1	F	Bilateral TP cortical dysplasia	ERMARD, VOUS	moderate	3 y	FBTCS	MultiFocal S-W	none	Physiological	VPA	LEV	no	none
2	M	P cortical dysplasia	NA	severe	2 y	FBTCS	MultiFocal S-W	none	Physiological	VPA	VPA, LEV, CLO	yes	ventriculomegaly
3	F	Complex DNET + FCD	NA	no	5 y	FBTCS	Multifocal S-W	none	Physiological	no	CBZ	no	none
4	M	Complex DNET + FCD	NA	no	12 y	FBTCS	MFED	none	Physiological	no	LEV	no	none
5	M	Subcortical heterotopia	TUBA1A, GPR 56, TUBA2B: not pathogenetic	moderate	3 y	FBTCS	Multifocal S-W	no	IUGR, anhydramnios	no	VPA	no	ventriculomegaly
6	F	Lissencephaly type V	NA	severe	14 mo	FS	bilater S-W, asymmetric bg	none	threatened spontaneous abortion	none	PHE	no	millimetric calcifications and ipodensity of periventricular white matter
7	M	Lissencephaly	array-CGH: not pathogenetic	severe	5 mo	FBTCS	Syncronous bursts alternating with isoelectric bg	died at 3 y old for respiratory failure	HSV1 infection during the first trimester	VPA, ACTH	TPA, VPA LEV	yes	triventricular hydrocephalus
8	M	Lissencephaly type I	POMGNT2, FAT4 VOUS	severe	2 mo	FS	Syncronous burstsalternating with isoelectric bg	none	IUGR	PHB	LEV	no	ventriculomegaly, hypoplasic vermis
9	F	Lissencephaly- pachygyria	karyotype, telomer analysis, MECP2: normal	moderate	4 mo	IS	hyps	none	FIVET, placental abruption	VGB	VPA. TPA	yes	ponto-cerebellar hypoplasia
10	F	O pachygyria	NA	severe	10 mo	FS	Bi-Occ S-W	kidney malformation	threatened spontaneous abortion I trimester	PHB	CLO, TPA	yes	ventriculomegaly
11	F	Bilateral F polymicrogyria	NA	severe	7 y	FBTCS	F S-W	aggressiveness, hypospadias	Physiological	VPA	LAC, LEV, CLO	yes	none
12	M	Bilateral FP polymicrogyria	NA	severe	20 days	FS	slow-bg	Ulcerative colitis	congenital CMV	PHB	VPA	no	ventriculomegaly, asimmetric frontal lobes, periventricular calcifications
13	M	Bilateral F polymicrogyria	array-CGH: not pathogenetic	severe	3 mo	FS	F-T S-W	none	Physiological	none	PHB	no	none
14	M	Bilateral PO polymicrogyria	NA	moderate	2 mo	FS	F-T S-W	cortical blindness	intrauterine twin death	none	PHB	no	none
15	F	Bilateral FP polymicrogyria	NA	severe	8 d	FS	F-P S-W Slow bg	none	congenital CMV infection	TPA	none (sz-free)	no	ventriculomegaly, periventricular calcifications
16	F	Bilateral F polymicrogyria	array-CGH: not pathogenetic	moderate	2mo	FBTCS	F S-W	Congenital hypotiroidism	Physiological	VPA	LEV, CBZ	yes	vermis hypoplasia
17	M	Unilateral schizencephaly	COL4A1: likely pathogenetic variant	severe	11mo	FS	Multifocal S-W	none	hypertransaminasemia	LEV	LEV, TPAC LO	yes	ventriculomegaly
18	F	Periventricular-nodular- heterotopia	HESX1, VOUS	no	15y	FS	Bi -Occ S-W	GH deficit, congenital hypothyroidism	threatened spontaneous abortion	no	LEV	no	none
19	F	Subcortical heterotopia	array-CGH: not pathogenetic	no	13 y	FS	F-T S-W	no	Physiological	no	TPA	no	none

Legend: M = male; F = female; y = years; mo = months; MCD = malformation of cortical development; VOUS = variant of uncertain significance; DD/ID=developmental delay/intellectual disability; NA = not available; C = central; F = frontal; P = parietal; T = temporal; R = right; L = left; FBTCS = focal to bilateral tonic clonic seizure; IS = infantile spasm; TS = tonic seizure; AtS = atonic seizure; AAS = atypical absence seizure; FS = focal seizure; multifocal S-W = multifocal spike-wave; SW = spike wave; slow bg = slow background; Bi-Occ = bilateral occipital; FL = frontal lobe; FCD = focal cortical dysplasia; CC = corpus callosum; hyps = hypsarrhythmia; DNET = dysembryoblastic neuroepithelial tumors; POMGNT2 = protein O-linked mannose N-acetylglucosaminyltransferase 2; FAT4 = FAT atypical cadherin 4; COL4A1 = collagen type IV alpha 1 chain; MECP2 = methyl-CpG binding protein 2; VPA = valproate; TPA = topiramate; LEV = levetiracetam; CBZ = carbamazepine; VGB = vigabatrin; PHB = phenobarbital; LAC = lacosamide; CLO = clonazepam; PKU = phenylketonuria.

## Data Availability

The data used to support the findings of this study may be released upon application to the corresponding author, who can be contacted at ppavone@unict.it.
